# Prediction of breeding values for group-recorded traits including genomic information and an individually recorded correlated trait

**DOI:** 10.1038/s41437-020-0339-3

**Published:** 2020-07-14

**Authors:** Xiang Ma, Ole F. Christensen, Hongding Gao, Ruihua Huang, Bjarne Nielsen, Per Madsen, Just Jensen, Tage Ostersen, Pinghua Li, Mahmoud Shirali, Guosheng Su

**Affiliations:** 1 Institute of Swine Science, Nanjing Agricultural University, Nanjing, 210095 China; 2 College of Animal Science and Technology, College of Veterinary Medicine, Zhejiang Agriculture and Forest Universiry, Hangzhou, 311300 China; 3 Department of Molecular Biology and Genetics, Center for Quantitative Genetics and Genomics, Aarhus University, 8830 Tjele, Denmark; 4 SEGES, Pig Research Centre, 1609 Copenhagen, Denmark

**Keywords:** Genetic markers, Animal breeding

## Abstract

Records on groups of individuals could be valuable for predicting breeding values when a trait is difficult or costly to measure on single individuals, such as feed intake and egg production. Adding genomic information has shown improvement in the accuracy of genetic evaluation of quantitative traits with individual records. Here, we investigated the value of genomic information for traits with group records. Besides, we investigated the improvement in accuracy of genetic evaluation for group-recorded traits when including information on a correlated trait with individual records. The study was based on a simulated pig population, including three scenarios of group structure and size. The results showed that both the genomic information and a correlated trait increased the accuracy of estimated breeding values (EBVs) for traits with group records. The accuracies of EBV obtained from group records with a size 24 were much lower than those with a size 12. Random assignment of animals to pens led to lower accuracy due to the weaker relationship between individuals within each group. It suggests that group records are valuable for genetic evaluation of a trait that is difficult to record on individuals, and the accuracy of genetic evaluation can be considerably increased using genomic information. Moreover, the genetic evaluation for a trait with group records can be greatly improved using a bivariate model, including correlated traits that are recorded individually. For efficient use of group records in genetic evaluation, relatively small group size and close relationships between individuals within one group are recommended.

## Introduction

Group records could be valuable for predicting breeding values (BVs) when traits are difficult or costly to measure on individuals, such as egg production or feed intake. Previous studies have shown negligible differences between the estimated variance components and considerable consistency in the ranking of BVs estimated from full-sib group records and from individual records for fish and laying hens (Nurgiartiningsih et al. 2004; Simianer and Gjerde 1991). Olson et al. (2006) proposed a model to use pooled records for predicting BVs of individuals in the group, and it was demonstrated that selection based on evaluations from group records can be very effective, particularly when the group size is small. Recently, Su et al. (2018) proposed a method that could appropriately handle multiple fixed and random effects (litter and pen effects) for estimation of variance components and prediction of BVs using group records with varying group sizes. Their results showed that the estimated variance components were consistent with those estimated from individual records, but with larger standard errors, and the accuracy of EBV from group records with size of 12 individuals reached up to 70% of the accuracy obtained from individual records.

In practice, individual recording is difficult for some traits such as feed intake, while easy for some of their correlated traits such as daily gain. Thus, it is expected that the accuracy of genetic evaluation for a trait with group records can be greatly improved by the information of a correlated trait with individual records using bivariate analysis.

Genomic prediction (Meuwissen et al. 2001) is a popular tool for estimating BVs. In the situation where not all animals are genotyped, the single-step genomic BLUP (ssGBLUP) is a good approach to estimate BVs accurately, since it simultaneously uses information from all phenotypes, pedigree, and markers (Aguilar et al. 2010; Christensen and Lund 2010; Legarra et al. 2009). Previous studies based on individual records have shown that genomic information greatly increases the accuracy of EBVs for complex traits. It can be hypothesized that genomic information will increase accuracy of EBVs predicted using group records. In addition, since genomic information can capture Mendelian sampling error, it can be suggested that genomic information could be more important for group records than for individual records in predicting BVs. However, using genomic information to estimate BVs based on group records has not been investigated.

The objectives of this study were to investigate the accuracy of genetic evaluation for traits with group records using (1) different proportions of genotyped animals (0, 30, and 100%), and (2) including a correlated individual-recorded trait in a bivariate model.

## Materials and methods

### Simulation of data

**Fig. 1:**
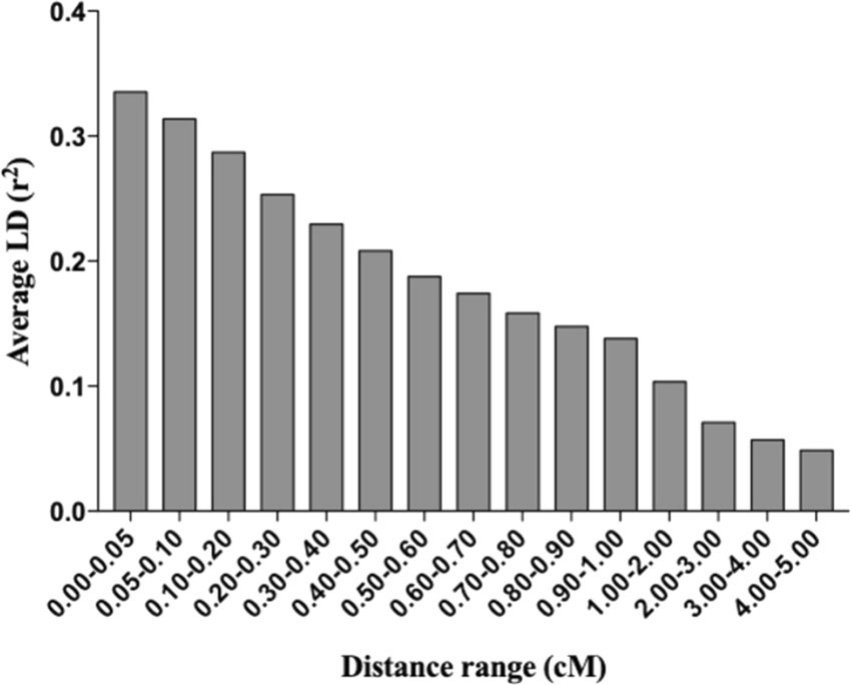
Linkage disequilibrium (LD) between markers in the last generation (8th) of the recent population. The x-axis displays the distance range of markers in the genome, expressed in centimorgan (cM); the y-axis displays the average LD, expressed in *r*^2^.

Estimation of variance components and prediction of BVs using group and individual records were evaluated using simulated data mimicking a pig nucleus population. The data were generated by QMSim (Sargolzaei and Schenkel 2009). Briefly, a historical population of 400 unrelated animals with equal sex ratio was generated and mated randomly for 300 generations with a constant size of 400 in each generation. To create the base population, 30 sires were selected randomly from the last historical generation and subsequently mated with 200 dams of the last historical generation to produce 1200 offspring with equal sex ratio (i.e., 600 males and 600 females, and litter size = 6). Then 600 dams (all 600 females) and 30 sires (randomly selected from 600 males) were chosen as the founder animals (generation 0) of the recent population. To create the phenotypic data, in each of the next eight nonoverlapped generations, 30 males and 600 females were randomly chosen as sires and dams that produced 600 litters (each sire mated to 20 dams randomly), and each litter comprised three males and three females. Litter size 6 is used to mimic the situation that about six pigs from each litter are tested, whereas the actual litter sizes in pigs are larger. The pattern of LD between the markers in the last generation of the recent population is shown in Fig. 1. The degree of LD in the simulated population was consistent with real livestock populations such as pig populations.

For genomic information, the simulated genome consisted of 18 chromosomes, each 100 centimorgans (cM) in length. Each chromosome included 3100 markers and 50 QTLs; all markers and QTLs were biallelic inz the first historical generation with random allele frequency. Markers and QTLs were all randomly distributed across the genome. The marker and QTL mutation rate were all 2.5e − 5 in the historical population. Only the loci with minor allele frequency larger than or equal to 0.01 were used to simulate the recent population. In total, 43,638 markers and 708 QTLs were segregating in the genome for the recent population (the parameter file for QMSim is available on request). The QTL allele effects for two traits (e.g., feed intake and daily gain) were sampled from a bivariate normal distribution with correlation 0.8, which corresponds to the genetic correlation between feed intake and daily gain in pigs (Hoque et al. 2009). The true BVs of trait 1 (*a*_1_) and trait 2 (*a*_2_) were defined as the sum of the QTL allele effects, and then the BVs were scaled to have the variances as the designed values (Table 1). The pen, litter, and residual effects for the two traits were sampled from a bivariate normal distribution with variances and covariance equal to those shown in Table 1.

**Table 1:** The variances and correlation coefficients for the simulation of data for two traits.

Variances	Trait 1 (*h*^2^ = 0.3)	Trait 2 (*h*^2^ = 0.25)	Correlation coefficient
Pen	10	40	0.3
Litter	10	40	0.3
Additive genetics	30	100	0.8
Residual	50	220	0.5

Only the last four generations (i.e., from generation 5 to generation 8, 14,400 individuals) of phenotypic and genomic data were used for analysis, and the pedigree was traced back to the base generation (generation 0) of the recent population. The pedigree contained 29,430 individuals in total. The last generation was used for validation, and two situations were considered. The first situation is that the phenotypic records of the last generation were kept (Valid_R), assuming that selection candidates had their own records (either individual or group record) at the time of selection; the other situation is that the phenotypic records of the last generation were removed (Valid_nR), assuming that selection candidates did not have their own records at the time of selection. The group record for trait 1 was defined as the sum of individual phenotypic records within one pen. The construction of pens was performed within the generation. Three different scenarios of group records concerning the size and structure of the group were investigated in this study (Table 2). (1) S_12_L_2 × 3_: a litter was divided into two sublitters of size 3 randomly and distributed into two pens; thus, a pen with 12 pigs included four random sublitters. (2) S_12_L_ran_: pigs in a litter were randomly distributed to different pens, and the number of pigs in a pen was up to 12. (3) S_24_L_2 × 3_: compared with the first scenario, the group size was set to 24, and thus a pen included pigs from 8 random sublitters. In practice, group sizes in a dataset are not always constant due to mortality, and different pen sizes and stocking densities between farms. To illustrate that the models in the paper can be used for such real situations also, 20% of the animals were randomly deleted (all breeding animals were kept) to create differences in group sizes. For each scenario, 50 replicates (each generated by one QMSim simulation) were generated and analyzed. The mean and standard deviation for the estimated parameters and accuracy of prediction from these 50 replicates were presented.

**Table 2:** Description of three different scenarios for trait 1.

Scenario	Pen size	Group composition
S_12__L_2 × 3_	4–12 Average 9.6	A litter was divided into two sublitters and distributed into two pens, each pen included four sublitters
S_12__L_ran_	4–12 Average 9.6	Randomly assigned individuals to pens, each pen contained up to 12 individuals
S_24__L_2 × 3_	12–24 Average 19.2	A litter was divided into two sublitters and distributed into two pens, each pen included eight sublitters

### Statistical analyses

Variance components were estimated using the average information-restricted maximum likelihood approach (Gilmour et al. 1995). BVs were predicted with the true variances using the best linear-unbiased prediction (BLUP) approach with the models presented below, based on data with no genotype information (Genotype_0), genotypes for all animals (Genotype_100), and genotypes for 30% of animals (Genotype_30), respectively. The 30% genotyped animals consisted of two parts, one was all the breeding animals (16.5%), and the other was animals randomly selected from the remaining animals (13.5%). All analyses were performed using the DMU release 5.4 (Madsen et al. 2006). In the prediction of BV in this study, to reduce the time of computation, variances in the models were not estimated, and the true variances were used instead. Accuracy and bias of BV prediction were used to assess the efficiency of prediction using group records versus individual records. Accuracy was defined as the correlation between predicted and true BV, and bias was measured as the regression coefficient of true BV on predicted BV. For fair comparisons, accuracies and bias were calculated for the three groups of animals: (1) all animals in the validation data (All), (2) the validation animals that were genotyped in the 30% genotyping scenario (Group I), and (3) the animals that were not genotyped in the 30% genotyping scenario (Group II).

### Univariate analysis

For trait 1, the variance component estimation for the first scenario (S_12_L_2 × 3_) and BV prediction for the three different scenarios were performed using PBLUP (for genotyping scenario Genotype_0), GBLUP (for Genotype_100), and ssGBLUP (for Genotype_30) methods.

When trait 1 was measured on individuals, the variance components and BVs were estimated using the following linear mixed model:

$${\boldsymbol{y}} = 1\mu + {\boldsymbol{Z}}_{\boldsymbol{l}}{\boldsymbol{l}} + {\boldsymbol{Z}}_{\boldsymbol{c}}{\boldsymbol{c}} + {\boldsymbol{Z}}_{\boldsymbol{a}}{\boldsymbol{a}} + {\boldsymbol{e}},$$

where ***y*** is the vector of individual records, *μ* is the overall mean, **1** is a vector of ones, ***l*** is the vector of litter effects, ***c*** is the vector of pen effects, ***a*** is the vector of additive genetic effects, ***e*** is the vector of residual effects, and ${\boldsymbol{Z}}_{\boldsymbol{l}},{\boldsymbol{Z}}_{\boldsymbol{c}},{\boldsymbol{Z}}_{\boldsymbol{a}}$ are the incidence matrices linking ***l***, ***c***, and ***a*** to ***y***. Litter effects reflect the common effects on littermates due to common environment before weaning, while pen effects represent the common environment effects on penmates during the test period. It was assumed that the random effects have the following distributions:

$${\boldsymbol{l}}\sim N\left( {0,{\boldsymbol{I}}\sigma _l^2} \right),{\boldsymbol{c}}\sim N\left( {0,{\boldsymbol{I}}\sigma _c^2} \right),{\boldsymbol{a}}\sim N\left( {0,{\boldsymbol{\Omega }}\sigma _a^2} \right),{\mathrm{and}}\;{\boldsymbol{e}}\sim N\left( {0,{\boldsymbol{I}}\sigma _e^2} \right),$$

where ***I*** is an identity matrix, ***Ω*** is the additive genetic relationship matrix that differs in different approaches (see ***A***, ***G***, ***H*** below), and $\sigma _l^2,\sigma _c^2,\;{\mathrm{and}}\;\sigma _a^2$ are the variances of litter effects, pen effects, and additive genetic effects, respectively.

When trait 1 was measured on groups (pens), the model for the group records can be written as shown by Su et al. (2018)

$${\boldsymbol{Ty}} = {\boldsymbol{T}}1\mu + {\boldsymbol{TZ}}_{\boldsymbol{l}}{\boldsymbol{l}} + {\boldsymbol{TZ}}_{\boldsymbol{c}}{\boldsymbol{c}} + {\boldsymbol{TZ}}_{\boldsymbol{a}}{\boldsymbol{a}} + {\boldsymbol{Te}},$$

where ***T*** is an incidence matrix that links individual records to particular groups with number of rows equal to the number of groups and number of columns equal to the total number of animals with records. In matrix ***T***, the element ***T***_*ij*_ = 1 if the *j*th animal belongs to the *i*th group, and otherwise ***T***_*ij*_ = 0. Matrix ***T*** functions as to sum variables for each level of a particular factor within a group (pen), such that ***Ty*** is the vector of group records, ${\boldsymbol{T1}}$ is the vector of group sizes, ***TZ***_*l*_, ***TZ***_***c***_, ***TZ***_*a*_ are incidence matrices linking effects to groups, and ***Te*** is the residual vector.

For PBLUP, which was used to analyze the data in the scenario where no individuals were genotyped, ***Ω*** = ***A***, where ***A*** is the relationship matrix built using pedigree information and considering inbreeding. For GBLUP, which was used to analyze the data in the scenario where all individuals were genotyped, ***Ω*** = ***G***, where ***G*** is the relationship matrix built using marker genotype information of all genotyped animals in the last four generations of the recent population, following method 1 by VanRaden (2008). The allele frequency used to construct the **G** matrix was directly calculated from the genotype data of these genotyped animals. For ssGBLUP, which was used to analyze the data in the scenario where 30% of individuals were genotyped, ***Ω*** = ***H***, where ***H*** is the relationship matrix built using both pedigree and genotype information. The ***H*** matrix was constructed according to (Aguilar et al. 2010; Christensen and Lund 2010; Christensen et al. 2012; Legarra et al. 2009)

$${\boldsymbol{H}} = \left[ {\begin{array}{*{20}{c}} {{\boldsymbol{G}}_{\boldsymbol{w}}} & {{\boldsymbol{G}}_{\boldsymbol{w}}{\boldsymbol{A}}_{11}^{ - 1}{\boldsymbol{A}}_{12}} \\ {{\boldsymbol{A}}_{12}^{\boldsymbol{T}}{\boldsymbol{A}}_{11}^{ - 1}{\boldsymbol{G}}_{\boldsymbol{w}}} & {{\boldsymbol{A}}_{22} + {\boldsymbol{A}}_{12}^{\boldsymbol{T}}{\boldsymbol{A}}_{11}^{ - 1}\left( {{\boldsymbol{G}}_{\boldsymbol{w}} - {\boldsymbol{A}}_{11}} \right){\boldsymbol{A}}_{11}^{ - 1}{\boldsymbol{A}}_{12}} \end{array}} \right].$$

The matrices ***A***_11_, ***A***_22_, and ***A***_12_ are submatrices of ***A*** containing relationships among genotyped, among nongenotyped, and between genotyped and nongenotyped animals, respectively. The matrix ${\boldsymbol{A}}_{12}^T$ is the transpose of ***A***_12_. Matrix ***G***_***w***_ is the genomic relationship matrix, including a proportion of ***A*** matrix (***A***_11_) for the subset of genotyped animals

$${\boldsymbol{G}}_{\boldsymbol{w}} = \left( {1 - {\boldsymbol{w}}} \right){\boldsymbol{G}} + {\boldsymbol{wA}}_{11},$$

where ***G*** is the matrix of the original genomic relationship matrix adjusted for the scale of ***A***_11_ using the method by Christensen et al. 2012, and the parameter ***w*** is set as 0.05 (Fragomeni et al. 2015).

### Bivariate analysis

For the bivariate model, trait 1 and trait 2 $$\left[ {\begin{array}{*{20}{c}} {{\boldsymbol{y}}_1} \\ {{\boldsymbol{y}}_2} \end{array}} \right]$$ were modeled jointly. The effects included in the bivariate model were the same as those in the univariate model. The random effects were assumed to have the following distribution:

$$\left[ {\begin{array}{*{20}{c}} {{\boldsymbol{a}}_1} \\ {{\boldsymbol{a}}_2} \end{array}} \right]\sim N\left( {0,\left[ {\begin{array}{*{20}{c}} {{\boldsymbol{\sigma }}_{{\boldsymbol{a}}_1}^2\;{\boldsymbol{\sigma }}_{{\boldsymbol{a}}_1{\boldsymbol{a}}_2}} \\ {{\boldsymbol{\sigma }}_{{\boldsymbol{a}}_1{\boldsymbol{a}}_2}\;{\boldsymbol{\sigma }}_{{\boldsymbol{a}}_2}^2} \end{array}} \right] \otimes {\mathbf{\Omega }}} \right),\;\left[ {\begin{array}{*{20}{c}} {{\boldsymbol{l}}_1} \\ {{\boldsymbol{l}}_2} \end{array}} \right]\sim N\left( {0,\left[ {\begin{array}{*{20}{c}} {{\boldsymbol{\sigma }}_{{\boldsymbol{l}}_1}^2\;{\boldsymbol{\sigma }}_{{\boldsymbol{l}}_1{\boldsymbol{l}}_2}} \\ {{\boldsymbol{\sigma }}_{{\boldsymbol{l}}_1{\boldsymbol{l}}_2}\;{\boldsymbol{\sigma }}_{{\boldsymbol{l}}_2}^2} \end{array}} \right] \otimes {\boldsymbol{I}}} \right),$$

$$\left[ {\begin{array}{*{20}{c}} {{\boldsymbol{c}}_1} \\ {{\boldsymbol{c}}_2} \end{array}} \right]\sim N\left( {0,\left[ {\begin{array}{*{20}{c}} {{\boldsymbol{\sigma }}_{{\boldsymbol{c}}_1}^2\;{\boldsymbol{\sigma }}_{{\boldsymbol{c}}_1{\boldsymbol{c}}_2}} \\ {{\boldsymbol{\sigma }}_{{\boldsymbol{c}}_1{\boldsymbol{c}}_2}\;{\boldsymbol{\sigma }}_{c_2}^2} \end{array}} \right] \otimes {\boldsymbol{I}}} \right),\left[ {\begin{array}{*{20}{c}} {{\boldsymbol{e}}_1} \\ {{\boldsymbol{e}}_2} \end{array}} \right]\sim N\left( {0,\left[ {\begin{array}{*{20}{c}} {{\boldsymbol{\sigma }}_{{\boldsymbol{e}}_1}^2\;{\boldsymbol{\sigma }}_{{\boldsymbol{e}}_1{\boldsymbol{e}}_2}} \\ {{\boldsymbol{\sigma }}_{{\boldsymbol{e}}_1{\boldsymbol{e}}_2}\;{\boldsymbol{\sigma }}_{{\boldsymbol{e}}_2}^2} \end{array}} \right] \otimes {\boldsymbol{I}}} \right).$$

When analyzing trait 1 with group records and trait 2 with individual records, our software was not able to handle the residual covariance between a group measurement on trait 1 and individual measurement on trait 2, and thus this covariance ${\boldsymbol{\sigma }}_{{\boldsymbol{e}}_1{\boldsymbol{e}}_2}$ was set to zero.

## Results

### Regression coefficients of true BV on predicted BV

For univariate analysis of all scenarios, the regression coefficients of true BV on the predicted BV using group and individual records are very similar. For bivariate analysis, although the residual covariance was forced to zero when using the bivariate model with group records for trait 1 and individual records for trait 2, the regression coefficients for predictions using group records for trait 1 were still similar to those using individual records for trait 1. All regression coefficients are around 1, indicating unbiased prediction for all scenarios, regardless of using group or individual records.

### Univariate analysis for a trait with group records

Table 3 presents the variance components for trait 1 estimated from the group and individual records in the first scenario (S_12_L_2 × 3_) using data of Genotype_0, Genotype_100, and Genotype_30. As expected, the standard deviations of the estimated variance components based on group records were much larger than those based on individual records. This indicates an information loss when using group records instead of individual records.

**Table 3:** The estimates of variance components (mean (SD) over 50 replicates) in the scenario S_12_L_2 × 3_ (group size = 12, individuals from four sublitters per pen) for trait 1 using univariate model based on group or individual records with different proportion of individuals having genotypes.

Record	Genotype proportion	Analysis	Pen variance ($\sigma _{c_1}^2$)	Litter variance ($\sigma _{l1}^2$)	Additive genetic variance ($\sigma _{a1}^2$)	Residual variance ($\sigma _{e1}^2$)
		Simulated	10	10	30	50
Group	100%	GBLUP	9.61 (4.37)	10.91 (6.64)	30.15 (5.28)	49.46 (37.63)
	30%	ssGBLUP	9.35 (4.45)	11.04 (6.60)	31.59 (7.00)	50.67 (37.34)
	0%	PBLUP	9.39 (4.55)	11.20 (6.77)	30.67 (7.90)	51.56 (39.88)
Individual	100%	GBLUP	9.98 (0.75)	9.96 (0.85)	30.11 (1.69)	50.15 (0.78)
	30%	ssGBLUP	9.96 (0.74)	9.46 (0.95)	33.41 (2.35)	48.57 (1.32)
	0%	PBLUP	9.85 (0.83)	9.94 (0.94)	30.85 (2.65)	50.03 (1.58)

For all scenarios, genotypic information resulted in a great increase in the accuracy of EBVs, for both group and individual records (Table 4). The highest accuracy was obtained when all animals were genotyped (Genotype_100), and the lowest one was obtained when no animals were genotyped (Genotype_0). Based on all validation animals, by increasing the genotyped proportion from 0 to 30%, the accuracy of the EBVs increased by 1–3 percentage points for Valid_R and 2–3 percentage points for Valid_nR. By increasing the genotyped proportion from 0 to 100% genotyped animals, the accuracy of the EBVs increased by 5–9 percentage points for Valid_R and by 6–11 percentage points for Valid_nR. For genotyping scenario Genotype_30, the accuracies for Group I were higher than for Group II, because Group I consisted of animals with genomic information. For Group I, by increasing the genotyped proportion from 0 to 30%, the accuracy of the EBVs increased by 4–6 percentage points for Valid_R and 4–8 percentage points for Valid_nR. By increasing the genotyped proportion from 0 to 100% genotyped animals, the accuracy of the EBVs increased by 5–9 percentage points for Valid_R and by 5–11 percentage points for Valid_nR. For Group II, by increasing the genotyped proportion from 0 to 30%, the accuracy of the EBVs increased by 1–2 percentage points for Valid_R and 1–3 percentage points for Valid_nR. By increasing the genotyped proportion from 0 to 100% genotyped animals, the accuracy of EBVs increased by 5–9 percentage points for Valid_R and by 6–11 percentage points for Valid_nR.

**Table 4:** The accuracy of EBV (mean (SD) of 50 replicates) in the scenario of (a) S_12_L_2 × 3_, (b) S_12_L_ran_, and (c) S_24_L_2×3_ for trait 1 using univariate model, based on group or individual records with different proportion of individuals having genotypes.

(a) Scenario^a^	Genotype proportion	Model	Animals^b^	Valid_R^c^			Valid_nR^c^		
				Group records	Indiv. records	*G/I* ratio^d^	Group records	Indiv. records	*G/I* ratio
S_12_L_2 × 3_	100%	GBLUP	All	0.63 (0.03)	0.89 (0.01)	0.71 (0.03)	0.47 (0.05)	0.81 (0.01)	0.58 (0.05)
			Group I	0.62 (0.05)	0.89 (0.01)	0.70 (0.04)	0.47 (0.07)	0.81 (0.02)	0.58 (0.06)
			Group II	0.63 (0.03)	0.89 (0.02)	0.71 (0.03)	0.47 (0.05)	0.81 (0.01)	0.58 (0.05)
	30%	ssGBLUP	All	0.56 (0.03)	0.77 (0.01)	0.73 (0.03)	0.40 (0.05)	0.64 (0.02)	0.63 (0.05)
			Group I	0.60 (0.05)	0.84 (0.02)	0.71 (0.04)	0.44 (0.06)	0.74 (0.03)	0.59 (0.05)
			Group II	0.56 (0.03)	0.76 (0.01)	0.73 (0.03)	0.39 (0.06)	0.62 (0.02)	0.64 (0.06)
	0%	PBLUP	All	0.54 (0.03)	0.74 (0.01)	0.73 (0.03)	0.37 (0.05)	0.56 (0.03)	0.66 (0.05)
			Group I	0.55 (0.04)	0.74 (0.03)	0.73 (0.03)	0.36 (0.06)	0.56 (0.04)	0.65 (0.05)
			Group II	0.54 (0.03)	0.74 (0.01)	0.73 (0.03)	0.37 (0.05)	0.56 (0.03)	0.66 (0.05)
(b) Scenario^e^									
S_12_L_ran_	100%	GBLUP	All	0.55 (0.04)	0.89 (0.01)	0.62 (0.04)	0.40 (0.05)	0.81 (0.01)	0.49 (0.05)
			Group I	0.55 (0.06)	0.89 (0.01)	0.62 (0.05)	0.40 (0.06)	0.81 (0.02)	0.50 (0.06)
			Group II	0.55 (0.04)	0.89 (0.01)	0.62 (0.04)	0.40 (0.05)	0.81 (0.01)	0.49 (0.05)
	30%	ssGBLUP	All	0.49 (0.04)	0.78 (0.01)	0.63 (0.04)	0.32 (0.05)	0.64 (0.02)	0.51 (0.05)
			Group I	0.52 (0.06)	0.84 (0.02)	0.61 (0.05)	0.36 (0.06)	0.74 (0.03)	0.49 (0.05)
			Group II	0.48 (0.04)	0.76 (0.01)	0.63 (0.04)	0.32 (0.05)	0.62 (0.02)	0.51 (0.05)
	0%	PBLUP	All	0.46 (0.04)	0.75 (0.01)	0.62 (0.04)	0.29 (0.06)	0.56 (0.03)	0.52 (0.06)
			Group I	0.46 (0.06)	0.74 (0.03)	0.62 (0.05)	0.29 (0.08)	0.56 (0.04)	0.51 (0.07)
			Group II	0.46 (0.04)	0.75 (0.01)	0.63 (0.04)	0.29 (0.06)	0.56 (0.03)	0.52 (0.06)
(c) Scenario^f^									
S_24_L_2 × 3_	100%	GBLUP	All	0.48 (0.04)	0.89 (0.01)	0.54 (0.04)	0.33 (0.06)	0.81 (0.02)	0.40 (0.05)
			Group I	0.48 (0.07)	0.89 (0.01)	0.53 (0.05)	0.32 (0.07)	0.81 (0.02)	0.40 (0.06)
			Group II	0.48 (0.04)	0.89 (0.01)	0.54 (0.04)	0.33 (0.06)	0.81 (0.01)	0.41 (0.05)
	30%	ssGBLUP	All	0.44 (0.04)	0.78 (0.01)	0.57 (0.04)	0.29 (0.07)	0.64 (0.02)	0.45 (0.06)
			Group I	0.47 (0.06)	0.84 (0.02)	0.55 (0.05)	0.31 (0.07)	0.74 (0.03)	0.42 (0.06)
			Group II	0.44 (0.04)	0.76 (0.01)	0.57 (0.04)	0.28 (0.07)	0.62 (0.02)	0.46 (0.06)
	0%	PBLUP	All	0.43 (0.04)	0.75 (0.01)	0.58 (0.04)	0.27 (0.07)	0.56 (0.03)	0.48 (0.06)
			Group I	0.43 (0.06)	0.74 (0.03)	0.58 (0.05)	0.27 (0.07)	0.56 (0.05)	0.48 (0.07)
			Group II	0.43 (0.04)	0.75 (0.01)	0.58 (0.04)	0.27 (0.07)	0.56 (0.03)	0.48 (0.06)

Accuracy was measured as correlation between true BV and EBV.

^a^S_12_L_2 × 3_, group size = 12, a litter into two groups.

^b^All: all animals; Group I: the animals which were genotyped in the 30% genotyping scenario; Group II: the animals which were not genotyped in the 30% genotyping scenario.

^c^Valid_R, validation for animals with records; Valid_nR, validation for animals without records.

^d^*G/I* ratio, ratio of accuracy for EBV predicted using group records to accuracy of EBV predicted using individual records.

^e^S_12_L_ran_, group size = 12, individuals random assigned to groups.

^f^S_24_L_2 × 3_, group size = 24, a litter into two groups.

Compared with grouping scenario 1, scenario 2 (S_12_L_ran_) led to lower accuracy due to weak relationships between individuals within the group, and the accuracy of the EBVs was about 86–88% of the accuracy in scenario S_12_L_2 × 3_. The increase in group size decreased the accuracy of the EBVs when using group records. The accuracy of the EBVs in Scenario S_24_L_2 × 3_ was about 76–80% of the accuracy in scenario S_12_L_2 × 3_.

The efficiency of group records in relation to individual records depends on scenarios. In grouping scenario S_12_L_2 × 3_, the accuracy of the EBVs predicted from group records in proportion to the accuracy of EBV predicted from individual records ranged from 71 to 73% for Valid_R, and from 58 to 66% for Valid_nR. In grouping scenario S_12_L_*ran*_, the percentages ranged from 62 to 63% for Valid_R, and from 49 to 52% for Valid_nR. In grouping scenario S_24_L_2 × 3_, the percentages ranged from 54 to 58% for Valid_R, and from 40 to 48% for Valid_nR. There was a tendency that the percentages decreased with the increasing proportion of animals being genotyped for valid_nR in S_12_L_2 × 3_ and S_24_L_2 × 3_.

### Bivariate analysis for a trait with group records and another with individual records

In the bivariate analysis, trait 1 (e.g., feed intake) was recorded either individually or at the group level, and trait 2 (e.g., daily gain) was recorded individually. The (co)variance components estimated from the group and individual records in scenario S_12_L_2 × 3_ are shown in Table 5. Similar to the outcome of the univariate model, variance components for trait 1 estimated from group records were consistent with those estimated from individual records, except for residual variances for which the estimates from group records were lower than (but not statistically significant) those from individual records. It was observed that the bivariate model with group records for trait 1 had a significant overestimation for pen covariance and a small overestimation for the other covariances. It seemed that by setting residual covariance between group records of trait 1 and individual records of trait 2 to zero, a part of residual covariance was moved to the estimate of pen covariance. Compared with the univariate model with group records for trait 1, the bivariate model greatly improved the accuracy of pen, litter, and residual variance estimates and slightly improved the accuracy of additive genetic variance estimate, as reflected by the smaller standard error (equivalent to the standard deviation in Table 5). However, the bivariate model did not improve the variance estimates for trait 1, which were recorded individually.

**Table 5:** The estimates of variance components (mean (SD) over 50 replicates) in scenario S_12_L_2 × 3_ (group size = 12, individuals from four litters per pen) for trait 1 and trait 2 using multivariate model based on group or individual records with different proportion of individuals having genotypes.

Record (trait 1 + trait 2)	Genotype proportion	Model	$\sigma _{c_1}^2$	$\sigma _{l_1}^2$	$\sigma _{a_1}^2$	$\sigma _{e_1}^2$	$\sigma _{c_2}^2$	$\sigma _{l_2}^2$	$\sigma _{a_2}^2$	$\sigma _{e_2}^2$	$\sigma _{c_1,c_2}$	$\sigma _{l_1,l_2}$	$\sigma _{a_1,a_2}$	$\sigma _{e_1,e_2}$
		Simulated	10	10	30	50	40	40	100	220	6	6	43.82	52.44
Trait 1 group + trait 2 individual	100%	GBLUP	10.23 (2.52)	11.25 (4.90)	30.40 (5.09)	43.76 (22.98)	39.40 (3.59)	40.33 (3.01)	98.49 (6.85)	219.08 (4.46)	11.16 (1.31)	6.09 (2.76)	44.79 (4.87)	0
	30%	ssGBLUP	10.23 (2.53)	10.86 (5.33)	32.56 (6.51)	42.74 (23.85)	39.31 (3.84)	38.19 (3.14)	109.51 (8.58)	214.18 (6.39)	10.97 (1.49)	5.56 (3.10)	48.79 (6.82)	0
	0%	PBLUP	10.34 (2.72)	11.47 (5.28)	31.14 (7.38)	42.73 (24.89)	39.40 (3.86)	39.84 (3.44)	103.43 (7.81)	218.22 (6.52)	11.18 (1.55)	6.36 (3.34)	45.95 (7.63)	0
Trait 1 individual + trait 2 individual	100%	GBLUP	9.88 (0.72)	10.02 (0.81)	29.74 (1.83)	50.00 (0.74)	39.28 (3.58)	40.32 (2.96)	98.48 (6.88)	219.17 (4.51)	5.83 (1.18)	5.94 (1.26)	43.47 (2.73)	52.43 (1.42)
	30%	ssGBLUP	9.90 (0.79)	9.56 (0.94)	32.41 (2.55)	48.67 (1.39)	39.29 (3.81)	38.32 (3.20)	109.14 (8.53)	214.30 (6.47)	5.86 (1.29)	5.30 (1.45)	47.46 (3.55)	50.20 (2.56)
	0%	PBLUP	9.90 (0.82)	10.06 (1.00)	30.29 (2.84)	50.08 (1.65)	39.35 (3.86)	39.92 (3.61)	102.73 (7.55)	218.60 (6.47)	5.88 (1.36)	5.96 (1.57)	44.59 (3.81)	52.17 (2.83)

$\sigma _{c_i}^2$, $\sigma _{l_i}^2$, $\sigma _{a_i}^2$, and $\sigma _{e_i}^2$ were the pen variance, litter variance, additive genetic variance, and residual variance for trait *i*. $\sigma _{c_1,c_2}$, $\sigma _{l_1,l_2}$, $\sigma _{a_1,a_2}$, and $\sigma _{e_1,e_2}$ were the pen covariance, litter covariance, additive genetic covariance, and residual covariance between the two traits.

The accuracies of the EBVs for trait 1 obtained from the bivariate analysis are shown in Table 6. As expected, the EBV accuracies for trait 1 obtained from the bivariate model had a much higher accuracy (by 11–15 percentage points for Valid_R and 11–22 percentage points for Valid_nR) than those obtained from the univariate model, when using group records (Table 6). However, when using individual records of trait 1, the improvement of EBV accuracy obtained from the bivariate analysis was very small for this trait, compared with those obtained from the univariate analysis. Based on all validation animals, by increasing the genotyped proportion from 0 to 30%, the accuracy of the EBVs increased by 3 percentage points for Valid_R and 7 percentage points for Valid_nR. By increasing the genotyped proportion from 0 to 100% genotyped animals, the accuracy of the EBVs increased by 13 percentage points for Valid_R and by 21 percentage points for Valid_nR. For Group I, by increasing the genotyped proportion from 0 to 30%, the accuracy of the EBVs increased by 8 percentage points for Valid_R and 15 percentage points for Valid_nR. By increasing genotyped proportion from 0 to 100% genotyped animals, the accuracy of the EBVs increased by 12 percentage points for Valid_R and by 21 percentage points for Valid_nR. For Group II, by increasing the genotyped proportion from 0 to 30%, the accuracy of the EBVs increased by 2 percentage points for Valid_R and 6 percentage points for Valid_nR. By increasing genotyped proportion from 0 to 100% genotyped animals, the accuracy of the EBVs increased by 13 percentage points for Valid_R and by 20 percentage points for Valid_nR. It was also observed that the gain in accuracy of the EBVs from the bivariate model was negligible for trait 2, which were recorded individually (Table 7), in comparison with the univariate analysis.

**Table 6:** The accuracy of EBV (mean (SD) of 50 replicates) in scenario S_12_L_2 × 3_ (group size = 12, individuals from four litters per pen) for trait 1 using bivariate model, based on group and individual records with different proportion of individuals having genotypes.

Genotype proportion	Model	Animals^a^	Valid_R^b^			Valid_nR^b^		
			Group records	Indiv. records	*G/I* ratio^c^	Group records	Indiv. records	*G*/*I* ratio
100%	GBLUP	All	0.78 (0.01)	0.89 (0.01)	0.87 (0.01)	0.69 (0.03)	0.81 (0.01)	0.84 (0.03)
		Group I	0.78 (0.03)	0.89 (0.01)	0.87 (0.02)	0.69 (0.04)	0.82 (0.02)	0.84 (0.03)
		Group II	0.78 (0.01)	0.89 (0.01)	0.87 (0.01)	0.68 (0.03)	0.81 (0.01)	0.84 (0.03)
30%	ssGBLUP	All	0.68 (0.02)	0.78 (0.01)	0.88 (0.01)	0.55 (0.04)	0.64 (0.02)	0.86 (0.03)
		Group I	0.74 (0.03)	0.85 (0.02)	0.88 (0.02)	0.63 (0.04)	0.75 (0.03)	0.84 (0.04)
		Group II	0.67 (0.02)	0.76 (0.01)	0.88 (0.01)	0.54 (0.04)	0.62 (0.02)	0.86 (0.04)
0%	PBLUP	All	0.65 (0.02)	0.74 (0.01)	0.87 (0.01)	0.48 (0.04)	0.56 (0.03)	0.86 (0.04)
		Group I	0.66 (0.03)	0.75 (0.03)	0.88 (0.02)	0.48 (0.05)	0.56 (0.04)	0.85 (0.04)
		Group II	0.65 (0.02)	0.74 (0.01)	0.87 (0.01)	0.48 (0.04)	0.56 (0.03)	0.86 (0.04)

Accuracy was measured as correlation between true BV and EBV.

^a^All: all animals; Group I: the animals that were genotyped in the 30% genotyping scenario; Group II: the animals that were not genotyped in the 30% genotyping scenario.

^b^Valid_R, validation for animals with records; Valid_nR, validation for animals without records.

^c^*G*/*I* ratio, ratio of accuracy for EBV predicted using group records to accuracy of EBV predicted using individual records.

**Table 7:** The accuracy of EBV (mean (SD) of 50 replicates) for trait 2 using univariate model and bivariate model, based on individual records with different proportion of individuals having genotypes.

Genotype proportion	Model	Animals^a^	Valid_R^b^		Valid_nR^c^	
			Univariate model	Bivariate model	Univariate model	Bivariate model
100%	GBLUP	All	0.86 (0.01)	0.86 (0.01)	0.77 (0.02)	0.78 (0.02)
		Group I	0.86 (0.01)	0.86 (0.01)	0.78 (0.02)	0.78 (0.02)
		Group II	0.86 (0.01)	0.86 (0.01)	0.77 (0.02)	0.78 (0.02)
30%	ssGBLUP	All	0.73 (0.01)	0.73 (0.01)	0.59 (0.02)	0.60 (0.02)
		Group I	0.80 (0.02)	0.81 (0.02)	0.70 (0.03)	0.70 (0.03)
		Group II	0.72 (0.01)	0.72 (0.01)	0.57 (0.02)	0.57 (0.02)
0%	PBLUP	All	0.70 (0.01)	0.70 (0.01)	0.52 (0.03)	0.52 (0.03)
		Group I	0.70 (0.02)	0.70 (0.02)	0.52 (0.04)	0.52 (0.03)
		Group II	0.69 (0.02)	0.70 (0.01)	0.52 (0.03)	0.52 (0.03)

Accuracy was measured as correlation between true BV and EBV.

^a^All: all animals; Group I: the animals that were genotyped in the 30% genotyping scenario; Group II: the animals that were not genotyped in the 30% genotyping scenario.

^b^Valid_R, validation for animals with records; Valid_nR, validation for animals without records.

## Discussion

In this study, the model for group records by Su et al. (2018) was extended to utilize genomic information and multiple-trait analysis. Three different scenarios concerning the structure and size of the group were investigated. The results showed that genotypic information greatly increased the accuracy of BV prediction, using both group and individual records. Still, prediction using individual records got more benefit from genomic information than prediction using group record did. The bivariate analysis with a correlated trait having individual records greatly increased the accuracy of EBVs for the target trait with group records, but negligible for the target trait with individual records.

### Efficiency of group records for prediction of individual BVs

In this study, the accuracy of EBVs from group records using a pedigree-based univariate model ranged from 0.43 to 0.54 for Valid_R and from 0.27 to 0.37 for Valid_nR, depending on the structure and size of the group, corresponding to 58–73% for Valid_R and 48–66% for Valid_nR of the accuracy of EBV obtained from individual records. The results are consistent with previous studies by Biscarini et al. (2008) based on data of laying hens, and Cooper et al. (2010) based on beef cattle. The results indicate that group records are very useful for genetic evaluation of traits that are difficult or costly to record individually, such as feed intake, egg production, etc.

Many studies have concluded that closer relationships between individuals in each group results in more accurate EBVs (Olson et al. 2006; Peeters et al. 2013; Su et al. 2018). Consistent results were obtained in our study. Compared with assigning four sublitters to one pen with up to 12 animals, randomly assigning individual pigs to a pen considerably reduced the accuracy of the EBVs from group records. This indicates that to use group records for genetic evaluation efficiently, the genetic relationship between individuals within a group should be taken into consideration, when grouping individuals.

Another important factor affecting the efficiency of group records for estimating BVs is group size. The larger the group size is, the more the information in the data is reduced when replacing individual observations with a single group record. Su et al. (2018) demonstrated a pattern that the accuracy of EBV decreased with increasing group size. In our study, the accuracy of EBVs from pooled data for the group with 24 individuals was about 76–80% for Valid_R and 70–74% for Valid_nR of accuracy for the group with 12 individuals. Considering feed intake in pigs, a group record is the total feed intake of the pigs that share the same feeder. When a pen is equipped with one feeder, a pen is a group, while when two pens are equipped with one common feeder, two pens are a group. Obviously, to use group records for estimating BVs for feed efficiency, one feeder for one pen is a better strategy.

### Improving the accuracy of EBV from group records by genomic information

In the present study, for the first time, genomic prediction using group records was investigated. A number of previous studies have confirmed that genomic prediction is more accurate than pedigree-based prediction based on data of individual records (Guo et al. 2015; Lund et al. 2011; Su et al. 2010; VanRaden et al. 2009). The results from the current study showed that genomic information greatly increases the accuracy of EBVs using group records. The amount of improvement increased with the proportion of animals having genotypes. With 30% of animals being genotyped, the gains were observed not only for genotyped animals, but also for nongenotyped animals. For genotyped animals in the 30% scenario, the accuracy of the EBVs was 94–98% for Valid_R and 89–97% for valid_nR of the accuracy when all animals are genotyped. The results suggest that it could be a good strategy to genotype 30% of animals with likely selection candidates being genotyped, since this strategy can reach a prediction accuracy for genotyped animals close to the accuracy when all animals are genotyped, but reduces genotyping cost greatly. Similar results were reported by (Henryon et al. 2014).

The gain from genomic prediction, compared with conventional pedigree-based BLUP prediction (Genotype_0), was larger in scenario S_12_L_ran_, where animals in each group had a relatively weak average genetic relationship, compared with scenario S_12_L_2 × 3_, where animals in each group had a high average genetic relationship. Many previous studies have shown that the accuracy of genomic prediction decreases with the reduction of genetic relationships between animals (Gao et al. 2013; Habier et al. 2010; Wu et al. 2015). On the other hand, it has been reported that the gain from genomic prediction, compared with conventional pedigree-based BLUP prediction, actually increases when the genetic relationship between reference and test animals is weak (Daetwyler et al. 2013; Habier et al. 2010; Wu et al. 2015). This is because genomic prediction models have the advantage of using population LD information to capture both the Mendelian segregation and the genetic links through unknown common ancestors (Su et al. 2012). It was observed that the gain from genomic information was related to the relationship between animals within the group. Compared with pedigree-based BLUP, GBLUP with all animals being genotyped increased the accuracy of EBVs by 9 percentage points for Valid_R and by 10 percentage points for Valid_nR in scenario S_12_L_2 × 3_, by 9 percentage points for Valid_R and by 10 percentage points for Valid_nR in scenario S_12_L_ran_. Similarly, compared with scenario S_12_L_2 × 3_, the accuracy of the EBVs in scenario S_12_L_ran_ decreased by 14% for Valid_R and by 22% for Valid_nR when using pedigree-based BLUP prediction, while by 12% for Valid_R and by 15% for Valid_nR when using GBLUP prediction. The results indicate that when the relationship between animals in the same group is weak, genomic information becomes relatively more valuable for estimating BVs from group records.

Using pedigree-based BLUP with group records, it is impossible to distinguish between full sibs within the same group, and thus these full sibs obtain the same EBV. With genomic information, it allows the full sibs within the same group to have different EBVs because the full sibs have different genotypes. Therefore, it was hypothesized that genomic information could benefit group records more than individual records for prediction of BVs. However, the results of the present study reject that hypothesis. In fact, there was a tendency that the accuracy of EBVs predicted from group records in proportion to the accuracy of EBVs predicted from individual records decreased with the increasing number of animals being genotyped, especially for valid_nR in S_12_L_2 × 3_ and S_24_L_2 × 3_. Similarly, it was found that genomic information did not largely improve the prediction of the social genetic effect (B. G. Poulsen, personal communication, March 6, 2020). The possible reason could be that, with individual records, genotype information is used more efficiently for predicting marker effect than with group records. This could be because individual genotype information links to group records when using group records. In contrast, individual genotype information directly links to the own individual record.

### Improving the accuracy of EBV from group records by using the information on correlated traits with individual records

When BVs were predicted using a bivariate model with group records for trait 1 and individual records for trait 2, accuracies of the EBVs for trait 1 increased considerably. Compared with univariate analysis, the accuracy of the EBVs from group records using the bivariate analysis increased by 11–15 percentage points for Valid_R and by 11–22 percentage points for Valid_nR. The large increase in accuracy was due to the high genetic correlation with the correlated trait and the correlated trait having individual records. In the case of no phenotypic information for trait 1, the BV can be indirectly predicted using trait 2. According to the accuracy of trait 2 using the univariate model (Table 7) and the genetic correlation of 0.8 between trait 1 and trait 2, the accuracies of the indirectly predicted BV for trait 1 were 0.68, 0.58, and 0.56 for Valid_R and 0.62, 0.47, and 0.42 for Valid_nR using data of trait 2 with 100%, 30%, and none of the individuals having genotypes, respectively. The accuracies were much higher than the EBV obtained directly from group records using the univariate model, but lower than the EBV obtained directly from group records using the bivariate model.

The results presented above were for the situation with a high genetic correlation. To investigate the sensitivity to the degree of genetic correlation, we carried out an extra study in which the genetic correlation coefficient was changed from 0.8 to 0.5. In such a case, the accuracies of indirectly predicted BVs for trait 1 using data of trait 2 were 0.43, 0.37, and 0.35 for Valid_R and 0.39, 0.30, and 0.26 for Valid_nR in scenarios of 100%, 30%, and none of the individuals having genotypes, respectively. The accuracies were much lower than the accuracies of EBV of trait 1 obtained directly from group records using the univariate model. Correspondingly, the benefit in accuracies of the EBVs for trait 1 with group records from the bivariate analysis was also relatively small. The gain was 5, 3, and 4 percentage points for Valid_R and 7, 3, and 3 percentage points for Valid_nR using data with 100%, 30%, and none of the individuals having genotypes, respectively (Table 8). So the benefit in accuracy of the EBVs for a trait with group records obtained from including a correlated trait with individual records depends crucially on the genetic correlation.

**Table 8:** The accuracy of EBV (mean (SD) of 50 replicates) for trait 1 with 0.5 genetic correlation coefficient between trait 1 and trait 2 using bivariate model with true variance components, based on group and individual records with different proportion of individuals having genotypes.

Genotype proportion	Analysis	Animals^a^	Valid_R^b^	Valid_nR^b^
100%	GBLUP	All	0.68 (0.02)	0.54 (0.02)
		Group I	0.67 (0.03)	0.54 (0.03)
		Group II	0.68 (0.02)	0.55 (0.04)
30%	ssGBLUP	All	0.59 (0.02)	0.43 (0.03)
		Group I	0.64 (0.03)	0.49 (0.04)
		Group II	0.59 (0.03)	0.43 (0.03)
0%	PBLUP	All	0.58 (0.03)	0.40 (0.03)
		Group I	0.58 (0.04)	0.40 (0.04)
		Group II	0.58 (0.03)	0.40 (0.03)

^a^All: all animals; Group I: the animals that were genotyped in the 30% genotyping scenario; Group II: the animals that were not genotyped in the 30% genotyping scenario.

^b^Valid_R, validation for animals with records; Valid_nR, validation for animals without records.

On the other hand, when trait 1 had individual records, the gain in accuracy of EBVs from the bivariate model was very small in comparison with the univariate analysis. In this study, the phenotypic information of a trait with individual records is sufficiently large (large sample, a large group of full sibs and half-sibs), such that the added information of the correlated trait has little value. For group records, the phenotypic information is much smaller compared with the information of individual records, and thus the correlated trait with individual records becomes important. Many studies have shown that a multiple-trait model is beneficial, especially for a trait with a small amount of phenotypic information (Guo et al. 2014; Jia and Jannink 2012; Tsuruta et al. 2011). Based on real data of beef cattle, Cooper et al. (2010) reported that using pen, total feed intake and individual daily gain was ~80% as effective as using individual feed intake and daily gain for feed intake for selection of feed efficiency, and was substantially more effective than indirect selection using daily gain alone. Our results also indicate that a bivariate model, including a correlated trait with individual records, is a good approach to improve the accuracy of EBVs for a trait with group records.

### (Co)variances for estimating BV using group records

Variances estimated from group records using the univariate model were unbiased and consistent with those estimated from individual records, but with large standard error. In the bivariate analysis with group records for one trait and individual records for the other trait, our software was not able to handle the correlated residuals. Thus, the covariance was set to zero. Setting the residual covariance to zero could also be the reason for an underestimation of the residual variance and slight increase in the other variances. It needs a more sophisticated model and software to handle the residual covariance between a trait with group records and a trait with individual records. However, even though residual covariance was improperly forced to zero, the EBVs were unbiased. The present study also predicted BVs using the estimated (co)variance components instead of the true ones. The accuracy and unbiasedness of the resulting EBVs were close to those predicted using true (co)variances (results not shown). These results suggest that the BLUP model is robust for predicting BV using group records.

### Practical uses of group records

In this study, using group records seemed very promising for predicting BVs for difficult or costly-to-measure traits. Several studies have investigated the use of group records for genetic evaluation in real livestock populations. Biscarini et al. (2008) and Biscarini et al. (2010) used cage records for predicting BVs of egg production in laying hens. Cooper et al. (2010) used pen records for predicting BVs of feed intake in beef cattle. Orengo et al. (2009) and Piles and Sánchez (2019) used cage records for estimating genetic parameters and comparing genetic effects in rabbit populations. Sánchez et al. (2014) used pen-average records for predicting BVs of feed intake in pigs. Compared with the previous ones, this study included more random effects (pen and litter) and integrated genomic information in the statistical model. However, the group record-related research was rarely observed in the application of small ruminants, such as sheep. It has been shown that there is decreased feed intake in sheep when sheep are housed in groups of less than four animals (Penning et al. 1993). Malik et al. (1996) used the pen-average record of feed intake instead of individual record for the selection of growth and efficiency in lambs. Still, individuals within the same pen could not be distinguished. The results of this study may support a cost-efficient strategy for predicting BVs of feed intake in sheep. The results of this study may support further uses of group records for genetic evaluation.

## Conclusions

We conclude that group records are valuable for genetic evaluation of a trait that is difficult to record individually. Although genomic information can lead to a large increase in the accuracy of genetic evaluation for traits with group records, we did not confirm that the genotypic information is more important for group records than for individual records in predicting BVs. In addition, genetic evaluation for a trait with group records can be further improved by a multiple-trait model, including correlated traits with individual records. Finally, for efficient use of group records in genetic evaluation, a relatively small group size and close relationships between individuals within one group are needed.
