# Correction: Prediction of breeding values for group-recorded traits including genomic information and an individually recorded correlated trait

**DOI:** 10.1038/s41437-020-00375-9

**Published:** 2020-09-22

**Authors:** Xiang Ma, Ole F. Christensen, Hongding Gao, Ruihua Huang, Bjarne Nielsen, Per Madsen, Just Jensen, Tage Ostersen, Pinghua Li, Mahmoud Shirali, Guosheng Su

**Affiliations:** 1 Institute of Swine Science, Nanjing Agricultural University, Nanjing, 210095 China; 2 College of Animal Science and Technology, College of Veterinary Medicine, Zhejiang Agriculture and Forest Universiry, Hangzhou, 311300 China; 3 Department of Molecular Biology and Genetics, Center for Quantitative Genetics and Genomics, Aarhus University, 8830 Tjele, Denmark; 4 SEGES, Pig Research Centre, 1609 Copenhagen, Denmark

**Keywords:** Genetic markers, Animal breeding

Correction to: *Heredity*

10.1038/s41437-020-0339-3

This article was originally published Online First without Open Access. After publication the author decided to opt for Open Choice and to make the article an Open Access publication. Therefore, the copyright of the article has been changed to © The Author(s) 2020 and the article is forthwith distributed under the terms of the Creative Commons Attribution 4.0 International License, which permits use, sharing, adaptation, distribution and reproduction in any medium or format, as long as you give appropriate credit to the original author(s) and the source, provide a link to the Creative Commons license, and indicate if changes were made. The images or other third party material in this article are included in the article’s Creative Commons license, unless indicated otherwise in a credit line to the material. If material is not included in the article’s Creative Commons license and your intended use is not permitted by statutory regulation or exceeds the permitted use, you will need to obtain permission directly from the copyright holder. To view a copy of this license, visit http://creativecommons.org/licenses/by/4.0/.

